# EXPLORING SEXUAL HEALTH AND HIV COMMUNICATION BETWEEN HEALTHCARE PROVIDERS AND OLDER PATIENTS

**DOI:** 10.1093/geroni/igz038.2021

**Published:** 2019-11-08

**Authors:** Erin L Robinson, Karla Washington, Geuhye Park, Laneshia Conner, Tara Lusby

**Affiliations:** 1 University of Missouri, School of Social Work, Columbia, Missouri, United States; 2 University of Missouri School of Medicine, Department of Family and Community Medicine, Columbia, Missouri, United States; 3 University of Missouri School of Social Work, Colu, Missouri, United States; 4 Spalding University School of Social Work, Louisville, Kentucky, United States

## Abstract

While HIV and aging is a complex issue, healthcare providers can play a vital role in education and screening among older patients. Therefore, this study aimed to better understand the frequency, content, and barriers/facilitators of provider communication with older patients around sexual health and HIV. One-on-one interviews were conducted with healthcare providers (N=18) and analyzed using Nvivo12. Providers reported that approximately 1-30% of their older patients show risk factors for STI’s. The majority of providers tailor their communication to older patients regarding sex/HIV, however they were divided in who initiates such conversations (themselves or the patient). Providers stated that standardized/routine health assessments help facilitate those conversations. Results indicate several barriers to conversations, such as age/gender differentials and time. Additional results will be discussed. Study findings can help guide provider education and screening of older adults regarding sexual health/HIV.

